# Characterization of the Canine Retinal Vasculature With Optical Coherence Tomography Angiography: Comparisons With Histology and Fluorescein Angiography

**DOI:** 10.3389/fnana.2021.785249

**Published:** 2021-12-13

**Authors:** Ana Ripolles-Garcia, Gordon Ruthel, Gui-Shuang Ying, Yineng Chen, Nicolas Cuenca, Gustavo D. Aguirre, William A. Beltran

**Affiliations:** ^1^Division of Experimental Retinal Therapies, Department of Clinical Sciences & Advanced Medicine, School of Veterinary Medicine, University of Pennsylvania, Philadelphia, PA, United States; ^2^Department of Pathobiology, School of Veterinary Medicine, University of Pennsylvania, Philadelphia, PA, United States; ^3^Department of Ophthalmology, Scheie Eye Institute, Perelman School of Medicine, University of Pennsylvania, Philadelphia, PA, United States; ^4^Department of Physiology, Genetics and Microbiology, University of Alicante, Alicante, Spain

**Keywords:** OCTA, canine retinal vasculature, vasculature quantification, vessel density (VD), vascular plexuses

## Abstract

**Purpose:** To present a methodology for quantification of the canine retinal vasculature imaged by optical coherence tomography angiography (OCTA) and validate this approach by comparison with fluorescein angiography (FA) and confocal imaging of retinal wholemounts labelled by immunohistochemistry (IHC).

**Methods:** Six normal adult dogs underwent retinal OCTA imaging in both eyes. The images extracted from the different microvascular plexuses at eight retinal locations spanning the central and mid-peripheral fundus were analyzed using the AngioTool software. FA was performed in one eye and was compared to the OCTA images. Six eyes from three dogs were processed by IHC to examine the retinal vasculature.

**Results:** A total of four retinal plexuses were identified by OCTA in the canine retina, and their density and topographical pattern varied with eccentricity. OCTA offered improved resolution over FA with the advantage of allowing imaging of the individual plexuses. Detection by OCTA of small vessels within the deep capillary plexus was possible and approached the level of resolution achieved with *ex vivo* imaging of the retinal vasculature by confocal microscopy/IHC. The plexuses herein described are analogous to human retinal vasculature.

**Conclusion:** OCTA can be used to image and quantify non-invasively the vascular retinal networks of the canine retina. We provide normative data in eight different retinal locations that can be imaged non-invasively with this technology. This could support analysis of retinal vascular changes associated with disease and following therapeutic intervention.

## Introduction

The retina is a complex and highly metabolic extracranial part of the central nervous system that requires a continuous and self-regulated blood supply (Harris et al., 1998; Yu and Cringle, 2001). In vascularised retinas, oxygenation is ensured by a well-organised retinal and choroidal vascular network. The choriocapillaris, a network of capillary vessels located under the retinal pigmented epithelium, is the main source of oxygen for the outer retina, and retinal vessels are the main supply for the inner retina (Michaelson, 1954). While choroidal vasculature has been maintained throughout evolution in vertebrates, the retinal vasculature pattern differs widely between species (Rochon-Duvigneaud, 1943). Based on the presence and distribution of this vasculature, four patterns have been described: euangiotic/holangiotic, merangiotic, paurangiotic and anangiotic (Leber, 1875; Schaepdrijver et al., 1989). The canine retina is classified as holangiotic as its blood vessels extend from the optic nerve head to the far periphery. In the temporally located *area centralis*, they converge toward the highly specialized fovea-like area (Peichl, 1992; Beltran et al., 2014).

However, the vasculature of the canine retina has not been thoroughly studied, and the location of the different vascular networks, as well as their nomenclature, remains unclear (Leber, 1875; Barrett, 1886; Mutlu and Leopold, 1964). In dogs, previous studies have imprecisely identified the presence of radially distributed blood vessels surrounding the optic nerve papilla, parallel to the nerve fibre layer (NFL) (Parry, 1953; Mutlu and Leopold, 1964). A second vascular network composed of major arterioles and venules has also been described and identified within the ganglion cell layer (GCL) (Parry, 1953; Mutlu and Leopold, 1964). Additionally, two capillary beds have been identified in the inner plexiform layer (IPL) (Schaepdrijver et al., 1989) and inner half of the outer plexiform layer (OPL) (Barrett, 1886; Parry, 1953; Mutlu and Leopold, 1964; Engerman et al., 1966; Schaepdrijver et al., 1989). However, the techniques used to describe the canine retinal microvasculature to date, such as vascular corrosion casting (Fischer and Slatter, 1978; Schaepdrijver et al., 1989), do not include structural analysis of the exact location of the vasculature in relation to the retinal layers. Furthermore, there are discrepancies between studies in the number of plexuses described (Michaelson, 1954). In contrast, four vascular plexuses have been identified and extensively described in the human retina (Chan et al., 2012).

Evaluation and quantification of the retinal vasculature is a valuable method to diagnose and monitor the progression of many retinal diseases that can result in blindness. This information serves as an essential diagnostic, prognostic, and therapeutic tool (Yao et al., 2020). Fluorescein angiography (FA) is currently the most widely applied technique in human and veterinary medicine for evaluation of retinal vessels *in vivo* (Gelatt et al., 1976; Cavallerano, 1996). Conventional FA has several disadvantages such as: suboptimal resolution in visualization of deeper retinal capillaries, and the need to use an intravenous contrast agent which may cause adverse systemic reactions (Kwiterovich et al., 1991; López-Sáez et al., 1998). Nonetheless, FA can detect increased vessel permeability evidenced by dye leakage, which is pathognomonic of blood-retinal barrier breakdown.

Optical coherence tomography angiography (OCTA) is a novel non-invasive technology that allows detailed visualization of retinal circulation, enabling the study of the vascular plexuses separately using their intrinsic motion decorrelation. The OCTA probabilistic full-spectrum amplitude decorrelation algorithm generates images based on the motion signal between a series of OCT b-scans acquired at the same position. The differences between the images acquired are due to erythrocyte movement in the blood vessels. By focusing on the movement, the algorithm is able eliminate all static sections of the scan area in order to generate a resulting image. This image is then further processed to produce the final *en face* angiogram (Coscas et al., 2016). Hence, it is able to detect vessel networks using their intrinsic motion decorrelation, thus avoiding the need for an intravenous dye (Jia et al., 2012).

While there are many different techniques and parameters utilized to quantify retinal vasculature, vessel density (VD) is the most widely used across studies (Pechauer et al., 2015). VD is generally defined as the percent area of an image that is occupied by blood vessels. However, the absolute values can vary widely between studies and there is a need for validation of novel OCTA-based approaches in canine retinas. Currently, the most recognized method of validating OCTA images is via direct comparison with retinal wholemounts processed for detection of the retinal vasculature by immunohistochemistry (IHC) (Yu et al., 2021). Ultimately, in order to be considered a reliable imaging technique, OCTA must recognize the majority of the vessels that are visualized by IHC.

The main objective of this study was to establish a methodology and normative data for retinal vasculature quantification by OCTA in canine eyes. We also aimed to validate our proposed methodology by comparison with FA and IHC. In order to achieve these goals, we investigated the anatomic features of the different retinal plexuses, their interconnections, and how their specific topographic patterns vary as a function of eccentricity from the optic nerve head.

## Materials and Methods

### Study Animals

The animals enrolled in the study were part of the Retinal Diseases Studies Facility research colony at the University of Pennsylvania. All the procedures were approved by the Institutional Animal Care and Use Committee of the University of Pennsylvania (IACUC# 804956).

The study population consisted of 6 normal, mesocephalic, mongrel dogs (12 eyes) with a mean age of 4.5 ± 1.3 years of age, and both genders were equally represented. All the animals underwent OCTA imaging in both eyes (OCTA module, Spectralis HRA + OCT2, version 6.9.4.0, Heidelberg Engineering Inc., Franklin, MA, United States) prior to FA being performed in one eye at the end of the procedure. Three animals, with both genders represented, where humanely euthanized with an overdose of a pentobarbital-based euthanasia solution (Euthasol, Virbac, Westlake, TX, United States) administered via intravenous injection, and their retinas were processed for IHC analysis, as previously described (Beltran et al., 2014).

For the *in vivo* imaging, the pupils were dilated with atropine sulfate 1%, tropicamide 1% (both drugs from Akorn Inc., Lake Forest, IL, United States) and phenylephrine 10% (Paragon Biotech, Portland, OR, United States). The dogs were anesthetised by intravenous propofol induction (2–6 mg/kg, Zoetis, Kalamazoo, MI, United States) and maintained with gas inhalation (isoflurane 2–3%, Akorn Inc., Lake Forest, IL, United States). Once positioned in sternal recumbency, the eyelids were kept open with an eye speculum and two stay sutures (Vicryl 4-0, Ethicon Inc., Somerville, NJ, United States). The stay sutures were placed in the conjunctiva 1–2 mm away from the limbus at the 2 and 10 o’clock position in order to gently manipulate the orientation of the visual axis as required to image the entire retina. The ocular surface was lubricated frequently with saline (Sodium Chloride 0.9%, ICU Medical, Inc., Lake Forest, IL, United States) and the anaesthetic plane was closely monitored via heart and respiratory rate, electrocardiography, blood pressure and oxygen saturation measurements.

### Optical Coherence Tomography Angiography Characterisation of the Retinal Vasculature

The retinal vascular characteristics were studied and quantified by obtaining 10-degree square OCTA images along the superior, inferior, temporal, and nasal retinal quadrants. For study purposes, 8 areas were preselected within the central and mid-peripheral retina (see Table 1). The mid-periphery scans corresponded to the most eccentric locations that could be reliably imaged with the OCTA unit. Indeed, the position of the globes in the orbit, and the shape of the canine skull precluded imaging of the peripheral retina. An additional area centered on the optic nerve head (ONH) was included for qualitative analysis only. The first scan was acquired in the *area centralis* and centered on the canine fovea-like area whose exact location was confirmed by anatomical features previously described including ONL thinning and a centrally converging vascular pattern (Supplementary Figure S1) (Beltran et al., 2014). Additionally, the orientation of the NFL on *en face* OCT shows a particular pattern bending around the very center of this cone-rich region (Supplementary Figure S1A), and this newly identified feature was used to accurately position the OCTA scanning window.

**TABLE 1:** Location of the areas of interest and their distance from the optic nerve.

Area of interest	Distance from optic nerve (mm) Mean ± SD
Optic nerve head	0
*Area centralis*	4.8 ± 0.18
Temporal mid-periphery	7.7 ± 1.49
Nasal centre	4.6 ± 0.59
Nasal mid-periphery	9.6 ± 1.05
Superior centre	5.9 ± 0.80
Superior mid-periphery	10.2 ± 1.27
Inferior centre	4.0 ± 0.63
Inferior mid-periphery	6.8 ± 1.33

Each imaged area was composed of volumetric scans that included 512 a-scans and 512 b-scans with 6 μm distance in between b-scans. This provided a lateral resolution of 5.75 μm/pixel and a depth resolution of 3.87 μm/pixel. In order to account for curvature bias, we calculated the retinal magnification factor based on the individual axial globe length and the following formula RMF = 2*π*(0.59*AGL)/360, as previously described (Beltran et al., 2014). The axial globe length was measured in all 12 eyes after each imaging session by ultrasound (Sonomed A-scan A1500, Sonomed-Escalon, Lake Success, NY, United States), and the mean ± SD was 21.2 ± 0.5 mm. By using this approach, we calculated that the 10 × 10 degree OCTA images corresponded in these dogs to a 2.2 ± 0.05 mm^2^ area. The exact area for each eye, was used to calibrate the AngioTool software (see below) and obtain accurate quantifications.

In order to individually image the vascular networks, the boundaries of the plexuses were identified and selected by adjusting the location where the algorithm is applied on the OCT b-scan, with the designated software (Heidelberg Eye Explorer, HEYEX). In order to define the most superficial individual retinal plexus the first boundary of the slab was placed on the ILM and the second segmentation line was manually displaced in depth until no vessels were captured on the *en face* OCTA. The upper boundary was then displaced to the endpoint of the previous one, and the same process was repeated to define the following plexus. In this way, we not only identified different plexuses but also described their location based on the retinal lamination seen by OCT.

Quantitative analysis was performed in all the areas of interest, with the exception of the ONH scan (Table 1). The OCT angiograms were extracted as .tiff files and then imported into the semiautomated vessel-analysis program AngioTool (Zudaire et al., 2011). This is a validated software, accessible in the public domain^1^ (0.6a version, National Cancer Institute, Bethesda, MD, United States). AngioTool processes images in order to obtain a set of quantitative parameters and has previously been used to quantify retinal vasculature in other species (Zudaire et al., 2011; Giannakaki-Zimmermann et al., 2016; Liu et al., 2017; Munk et al., 2017; Sun et al., 2021; Told et al., 2021). Once the angiogram is uploaded into AngioTool, the program allows adjustments to the vessel diameter and intensity to ensure that the resulting image matches the correct vessel profile. Once the analysis is completed, the program extracts the results in a working spreadsheet. For each analyzed slab, we selected among the AngioTool parameters: VD, Junction Density (JD), Average Vessels Length (AVL), and Lacunarity (LC). For more details, see Table 2.

**TABLE 2:** AngioTool parameters used to characterise the retinal vasculature.

Vascular parameter	Unit	Definition and interpretation
Vessel density (Vessels% area)	Vessels area/total area	Percentage of the sampled area that is covered by blood vessels. The values range from 0 to 100. Represents how densely vascularised a tissue is.
Junction density (Branching index)	Junctions/mm^2^	Number of vessel junctions normalised per unit area. Informs if there is angiogenesis or vessel loss over time.
Average Vessels length	mm	Mean length of all the vessels in the image. Represents the mean length of the vessels before they branch.
Lacunarity		Index for vascular structural non-uniformity. The higher the value, the more heterogeneous is the area analyzed. Low lacunarity indicates homogeneity in the sampled area.

### Validation of Optical Coherence Tomography Angiography Quantifications by Comparison With Fluorescein Angiography and Immunohistochemistry

Fluorescein angiography images were obtained from the last eye evaluated (left eye) in all dogs. The FA images were obtained via fluorescein injection into the cephalic vein (20 mg/kg of Fluorescein sodium 10%, AK-FLUOR, Akorn, Lane Forest, IL, United States). Several cSLO fundus photographs were acquired with a short wavelength laser (488 nm, Spectralis Blue Autofluorescence mode) at the completion of the venous phase defined by a homogeneous and complete filling of the retinal venules (Schaepdrijver et al., 1996). The images were acquired with a 30 degrees lens that provided a lateral resolution of 5.60 μm/pixel.

For wholemount preparation, the retinas were prepared as previously described (Beltran et al., 2014). Briefly, after 15 min of fixation in 4% paraformaldehyde, the neuroretina was separated from the RPE and the fixation continued for 4 h at 4°C. The tissues were incubated for 4 days at 4°C in a solution containing primary antibodies directed against Collagen IV (1:100, Millipore Sigma AB769, Burlington, MA, United States). Fluorochrome-labelled secondary antibodies (Alexa Fluor 488 and 568 dyes; 1:200, Thermo Scientific) and DAPI nuclear stain were applied for 24 h at 4°C. The retinas were imaged using a 2-photon confocal microscope (Leica SP8 Multiphoton, Leica Microsystems, Wetzlar, Germany) with a 20x (HCX APO L, 1.0 NA) water immersion objective lens. The captured images studied were composed of individual 0.5 mm^2^ fields, acquired from z stacks spanning from the ILM to the ONL. Z-stacks were acquired at 1.5 μm Z-steps in 1024 × 1024 pixel format at 400 Hz with a line average of 2. These settings offered a lateral resolution of 0.541 μm/pixel and axial resolution of 1.5 μm/pixel. The images were acquired, merged, and analyzed with Leica Application Suite (LAS X, 3D Visualisation Module).

The evaluation of the anatomical features of the plexuses by IHC was performed in three eyes by stitching tiled z-stacks, covering an area that extended from the ONH to the *ora serrata* in all four retinal quadrants. Using the 3D representation module of the Leica Application Suite, we measured the distance from the optic nerve to the area where each of the retinal vascular plexuses was no longer present, to identify the topographical distribution of each vascular network.

For quantitative analysis, the OCTA images were matched with those obtained with FA and IHC using a graphic editing software (Adobe Photoshop CC, Adobe Systems Inc., CA, United States) and by matching vascular landmarks in the areas of interest. For this purpose, the individual 0.5 mm^2^ fields on IHC were stitched to cover the same squared area that was previously imaged on OCTA in the eight areas of interest. From this merged three-dimensional stack, the plexuses were extracted by cropping the subset of data that contained the same vascular networks defined by OCTA. Once the individual stacks were extracted, the mass projection image was exported as a .tif file and used for quantification using AngioTool, as previously described.

### Statistical Analysis

All statistical analyses were performed using SAS v9.4 (SAS Institute, Cary, NC, United States). For all tests, two-sided *p* ≤ 0.05 was considered statistically significant.

The mean (standard deviation, SD) of each OCTA measurements from the different locations within each quadrant was calculated, and 95% normal limits were derived as mean ± 1.96*SD. For the statistical comparison of each OCTA measures between mid-periphery vs. central areas, generalised linear models were fitted and generalised estimating equations (GEE) were used to account for the inter-eye correlation for measures from two eyes of the same dog and correlation of measures at different locations from the same eye. In the GEE, the unstructured covariance structure was used.

For comparison of measures between imaging techniques (OCTA vs. FA and OCTA vs. IHC) that were measured in one eye per dog, paired *t*-tests were used to test their statistical significance.

## Results

### Optical Coherence Tomography Angiography Enables Identification of Four Vascular Plexuses in the Canine Retina

**FIGURE 1:**
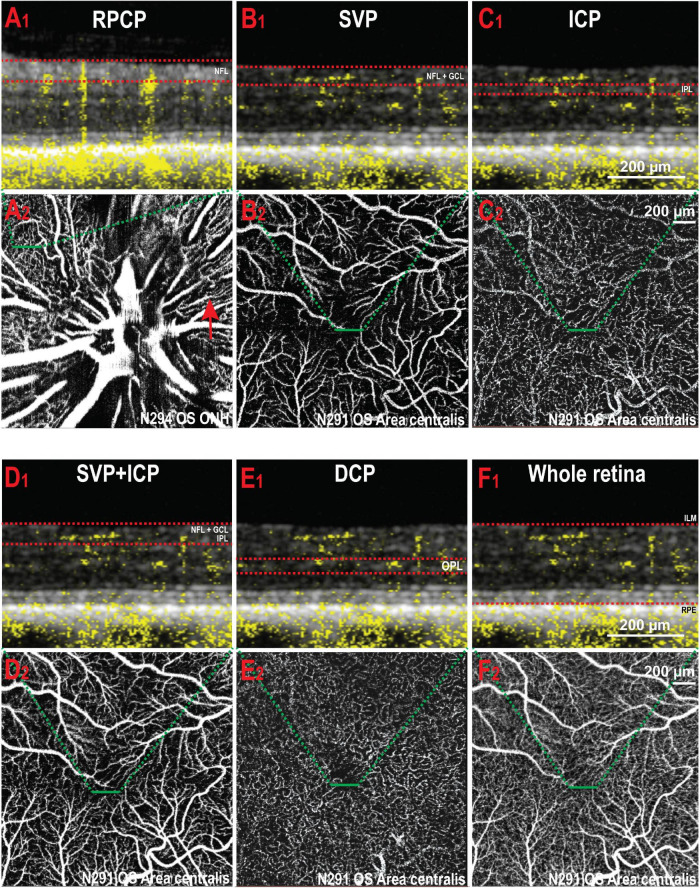
Illustration of the vascular plexuses of the canine retina imaged by OCTA. **(A1,2)** Radial peripapillary capillary plexus (RPCP). This plexus is only seen within the NFL (red arrow) around the optic nerve head. **(B1,2)** Superficial vascular plexus (SVP) in the *area centralis*. **(C1,2)** Intermediate capillary plexus (ICP) in the same area. **(D1,2)** SVP and ICP extracted together. **(E1,2)** Deep capillary plexus (DCP). **(F1,2)** OCT angiogram containing all the retinal vascular networks ( = whole retina thickness). The red dotted lines in panels **(A1–F1)** delineate the inner and outer boundaries of the slab selected to extract the angiograms shown in panels **(A2–F2)** (green dashed lines). NFL, nerve fibre layer; OS, left eye; ONH, optic nerve head; GCL, ganglion cell layer; IPL, inner plexiform layer; OPL, outer plexiform layer; ILM, inner limiting membrane; RPE, retinal pigment epithelium.

Manual segmentation in the OCTA module of the HEYEX software enabled precise localisation of individual retinal vascular plexuses within the retinal layers by concurrent visualisation of both the OCTA b-scan and the *en face* view of the angiogram (Figure 1). The most inner vascular network found within the NFL extended only into the peripapillary region and did not reach central areas. This plexus shared similarities with the human Radial Peripapillary Capillary Network (RPCP; Figures 1A1,2). The second vascular network spanned from the ILM to the GCL/IPL border, was formed by large arterioles, venules and their connecting capillaries, and branched from the ONH to the mid-periphery (most eccentric areas scanned by OCTA). It was analogous to the Superficial Vascular Plexus described in human retinas (SVP; Figures 1B1,2). We did not find an avascular zone in the canine SVP at the corresponding location of the canine fovea-like area, as is commonly seen in species with a foveal pit. The third vascular network was located within the IPL in proximity with the INL and contained capillaries which formed a homogeneous mesh. This vascular network was defined as the Intermediate Capillary Plexus (ICP; Figures 1C1,2). The SVP and ICP were seen in very close proximity, and they were frequently extracted together (Figures 1C1,2). The fourth and outermost plexus was located within the inner half of the OPL and formed a dense network of capillaries. This plexus was similar to the human Deep Capillary Plexus (DCP; Figures 1E1,2).

A previously undescribed feature seen in 9 out of 12 eyes consisted of a small focal area with a lower density of vessels in the DCP at the centre of the canine fovea-like area (Supplementary Figure S2A). We confirmed by IHC that this same anatomical feature was visible on retinal wholemounts in the DCP but not in the SVP (Supplementary Figure S2B).

### Vessel Density and Other Vascular Optical Coherence Tomography Angiography Parameters Vary With Retinal Eccentricity

**FIGURE 2:**
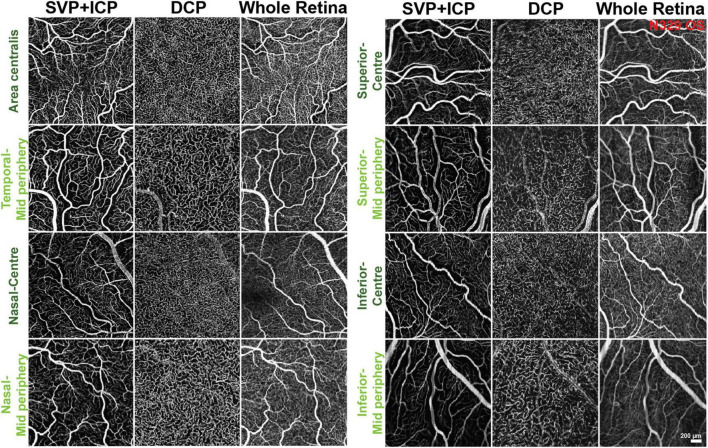
Representative OCTA images of the areas and slabs used for quantification. SVP, superficial vascular plexus; ICP, intermediate capillary plexus; DCP, deep capillary plexus.

Due to the presence of large vessels in the SVP that occasionally invade the ICP, individual differentiation and segmentation of these two plexuses was challenging. Consequently, both plexuses were extracted together in the same imaging slab to be quantified as a single image (Figures 1D1,2). The DCP was extracted next, and then the last slab, whose limitations were set at the ILM and RPE (whole retina, WR; Figures 1F1,2). Representative images from each area and slabs used for quantification are compiled in Figure 2.

The 95% confidence intervals of OCTA parameters for each slab (SVP + ICP, DCP and WR) were calculated, providing a range of reference values for this adult dog cohort (Table 3).

**TABLE 3:** Normal range of the vascular parameters in each retinal area.

Right eye (*N* = 6)									

95% Confidence limits		*Area centralis*	Temporal mid-periphery	Nasal centre	Nasal mid-periphery	Superior centre	Superior mid-periphery	Inferior centre	Inferior mid-periphery
SVP + ICP	VD	28.5–45.1	28.6–43.7	22.2–51.4	24.5–46.1	23.4–44.4	27.5–38.3	22.2–60.8	24.9–43.7
	JD	39.7–179.3	38.5–126.0	50.7–137.7	34.1–117.3	21.8–145.6	30.8–106.3	29.7–152.9	24.9–123.8
	AVL	0.10–0.66	0.17–0.58	0.12–0.66	0.08–0.68	0.01–0.80	0.15–0.64	–0.38 to 2	0.12–0.73
	LC	0.04–0.13	0.05–0.16	0.03–0.16	0.04–0.18	0.01–0.22	0.07–0.15	0.01–0.17	0.06–0.16
DCP	VD	39.1–49.8	35.6–51.0	30.4–49.6	26.0–50.3	27.0–53.8	34.1–47.1	38.4–47.5	27.8–43.2
	JD	125.9–262.0	81.3–280.2	87.8–231.8	40.3–224.6	60.8–279.7	52.7–217.6	121.5–214.0	70.6–146.6
	AVL	0.15–1.26	–0.3 to 1.66	0.06–0.77	–0.28 to 1.19	–0.33 to 1.55	0.10–0.84	0.09–1.22	0.05–0.57
	LC	0.03–0.07	0.03–0.09	0.03–0.11	0.01–0.16	0.00–0.15	0.04–0.09	0.05–0.07	0.05–0.14
WR	VD	39.0–47.5	32.0–48.8	32.8–45.3	33.8–44.6	27.8–47.1	25.9–59.4	31.2–46.9	25.8–48.5
	JD	129.5–258.0	93.2–210.9	110.0–210.1	103.3–209.7	47.0–227.9	89.0–220.3	106.2–187.0	60.9–219.7
	AVL	0.21–1.00	0.19–0.60	0.15–0.67	0.15–0.69	–0.01 to 0.75	–1.57 to 3.45	0.17–0.66	0.01–0.76
	LC	0.04–0.07	0.04–0.10	0.04–0.10	0.05–0.11	0.04–0.13	0.02–0.12	0.05–0.11	0.03–0.15

**Left eye (*N* = 6)**									

**95% Confidence limits**		***Area centralis***	**Temporal mid-periphery**	**Nasal centre**	**Nasal mid-periphery**	**Superior centre**	**Superior mid-periphery**	**Inferior centre**	**Inferior mid-periphery**

SVP + ICP	VD	35.1–46.5	31.7–44.2	30.2–49.3	32.0–40.2	21.7–62.2	31.9–42.4	31.7–43.2	24.1–46.3
	JD	81.7–216.0	55.6–117.6	37.1–141.4	77.9–109.2	62.3–144.7	72.6–116.3	52.8–142.1	18.9–112.6
	AVL	0.18–0.84	0.21–0.82	–0.06 to 1.19	0.17–0.72	–0.31 to 1.77	0.24–0.74	0.32–0.75	0.09–0.98
	LC	0.04–0.09	0.07–0.11	0.06–0.11	0.07–0.12	0.03–0.14	0.08–0.10	0.06–0.12	0.03–0.22
DCP	VD	42.1–49.0	33.3–49.6	35.4–51.8	30.8–50.6	39.8–48.4	37.8–45.9	38.5–48.3	27.9–44.6
	JD	139.5–281.7	81.3–221.6	65.3–333.9	51.6–254.2	140.3–232.2	125.3–177.5	114.7–235.3	68.0–162.1
	AVL	–0.09 to 1.74	–0.21 to 1.45	–0.36 to 1.77	–0.58 to 1.92	0.18–1.06	0.31–0.83	0.41–0.81	0.08–0.67
	LC	0.04–0.06	0.03–0.10	0.03–0.08	0.03–0.11	0.04–0.07	0.05–0.08	0.04–0.08	0.04–0.14
WR	VD	40.7–48.6	33.0–47.9	36.8–43.7	35.5–49.0	29.3–48.8	36.6–48.2	30.7–48.5	27.8–45.6
	JD	144.3–278.9	96.1–207.6	133.7–206.9	117.2–245.6	72.5–193.5	109.0–251.7	96.7–220.4	69.0–210.8
	AVL	0.28–1.14	0.26–0.63	0.34–0.49	0.22–0.92	0.09–0.71	0.27–0.82	0.15–0.79	0.06–0.64
	LC	0.04–0.06	0.03–0.12	0.06–0.08	0.03–0.10	0.04–0.13	0.04–0.09	0.03–0.11	0.03–0.17

*SVP, superficial vascular plexus; ICP, intermediate capillary plexus; DCP, deep capillary plexus; WR, whole retina; VD, vessel density (%); JD, junction density (number of junctions/mm^2^); AVL, average vessel length (mm); LC, lacunarity.*

**FIGURE 3:**
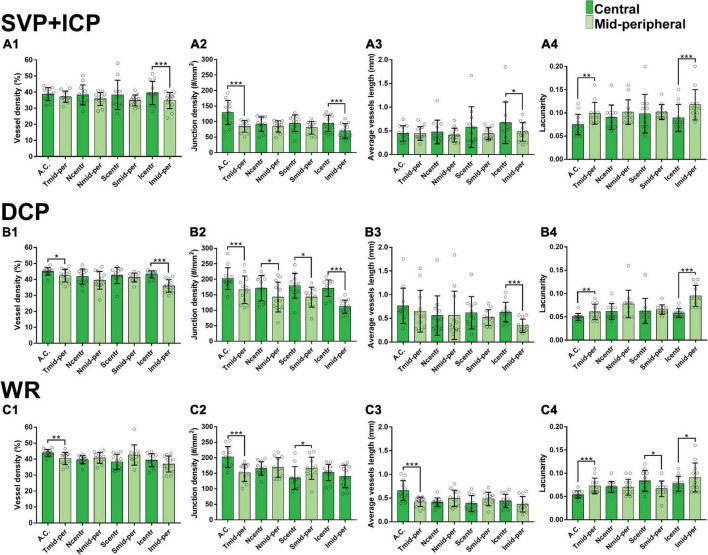
Topographical quantification of vascular plexuses by OCTA in the canine retina. The vessel density **(A1,B1,C1)**, junction density **(A2,B2,C2)**, average vessel length **(A3,B3,C3)** and lacunarity **(A4,B4,C4)** values are provided for the superficial vascular plexus and intermediate capillary plexus (SVP+ICP, **A1–4**), deep capillary plexus (DCP, **B1–4**) and whole retina slabs (WR, **C1–4**). A.C., *area centralis*; T, temporal; N, nasal; S: superior; I, inferior; mid-per, mid-periphery. Results are shown as a mean ± SD (*n* = 12). Paired *t*-test: *p ≤ 0.05, **p ≤ 0.01, ***p ≤ 0.001.

Along each quadrant, the VD was consistently lower in the mid-periphery in comparison to more central areas. This was observed for both the SVP + ICP and the DCP, although not always reaching statistical significance (Figures 3A1,B1,C1). JD values followed a similar topographical pattern, being higher in the more central areas than in the mid-periphery. This reached statistical significance in all four quadrants of the DCP (Figures 3A2,B2,C2). AVL results also showed a similar topographical trend, with mid-peripheral vessels being shorter due to decreased tortuosity (Figures 3A3,B3,C3). Finally, LC values were found to be higher in mid-peripheral than in central areas, suggesting increased vascular heterogeneity toward the periphery (Figures 3A4,B4,C4).

Analysis of the quality of the OCTA scans using signal to noise ratio values provided by the Spectralis software (HEYEX) showed that the best images were acquired in the *area centralis* and the poorest in the inferior mid-periphery. For more details see Supplementary Table S1.

### Optical Coherence Tomography Angiography Supersedes Fundus Fluorescein Angiography at Detecting Small Vascular Networks

Fluorescein angiography images were acquired at the late venous phase, which was seen at 18 ± 4.7 s after contrast agent injection. Since FA does not allow visualisation of individual vascular networks, the images were compared to the OCTA image extracted from the combination of all the slabs (WR). Qualitatively, OCT angiograms had a greater ability to resolve smaller vascular branches and capillaries than FA (Supplementary Figure S3A) and this was confirmed quantitatively when values of vascular parameters (VD, JD, AVL, and LC) were compared between the two techniques (Supplementary Figure S3B).

### Immunohistochemistry Validates Optical Coherence Tomography Angiography as a Reliable Technique to Image Retinal Vessels

**FIGURE 4:**
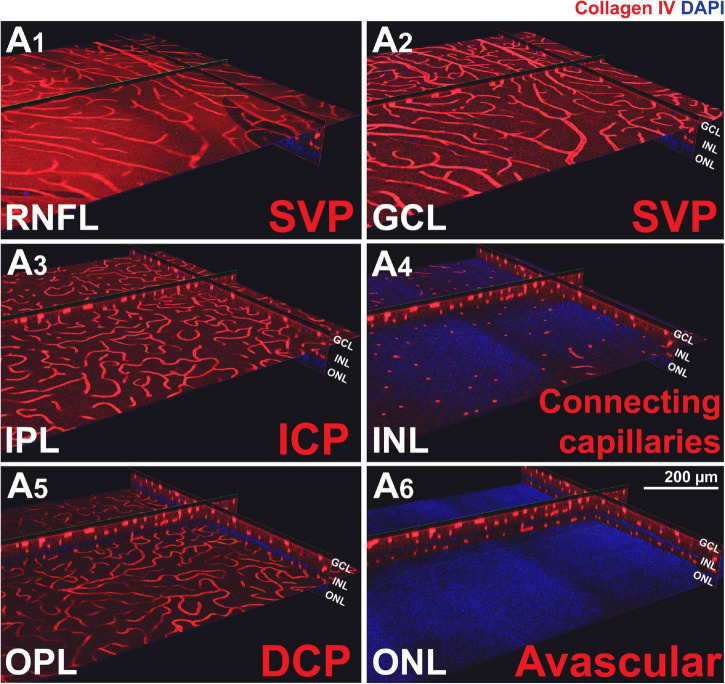
Canine retinal vascular plexuses imaged by 2-photon/confocal microscopy/IHC. **(A1,2)** *En face* and cross-sectional visualisation of the superficial vascular plexus (SVP), **(A3)** the intermediate capillary plexus (ICP), and **(A5)** the deep capillary plexus (DCP). **(A4)** The INL contains connecting capillaries from the ICP to the DCP. **(A6)** The ONL is avascular.

**FIGURE 5:**
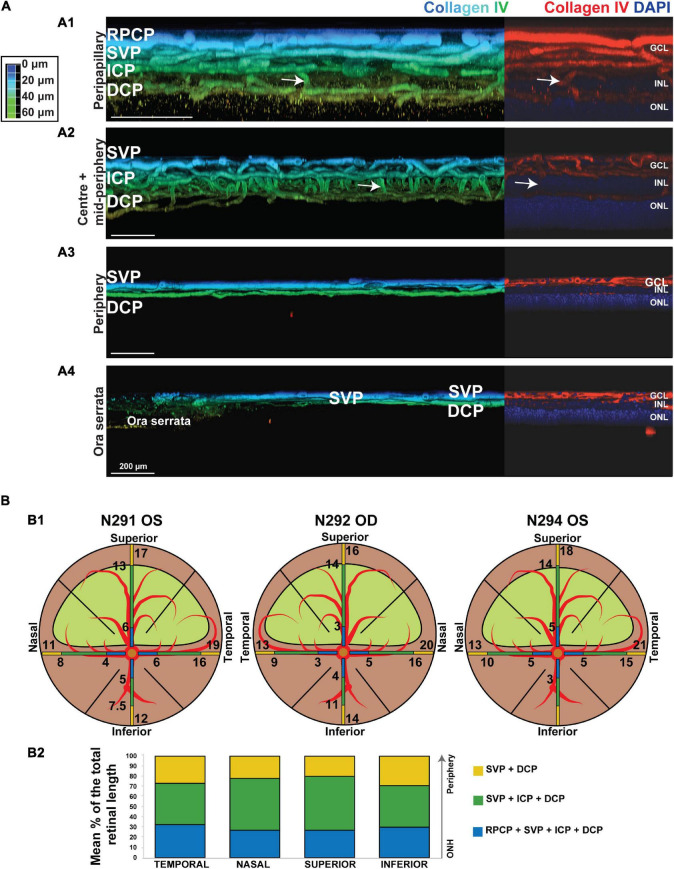
Topographical analysis by IHC of the vascular networks on canine retinal wholemounts. **(A)** Pseudocolored three-dimensional representation of the vascular plexuses imaged by 2 photon/confocal microscopy at four locations extending from the peripapillary region to the *ora serrata*. In the peripapillary region **(A1)**, all four plexuses are identified (RPCP, radial peripapillary capillary plexus; SVP, superficial vascular plexus; ICP, intermediate capillary plexus; DCP, deep capillary plexus). In the central and mid-peripheral retina **(A2)**, the RPCP is no longer seen. At the periphery **(A3)** only the SVP and DCP remain, and both merge at the far periphery close to the *ora serrata* **(A4)**. The right-side portion of each panel shows localisation of the Collagen IV vascular marker (in red) and DAPI (in blue) for improved visualisation of the retinal layers in which the plexuses are located. **(B)** Graphic representation of the topographical extension from the ONH of each vascular plexus measured in three individual retinal wholemounts **(B1)** and averaged as percentage of retinal length (ONH to *ora serrata* distance) in **(B2)**. Numerical values shown on the retinal diagrams **(B1)** represent the distance from the centre of the ONH to the point where a change in the number of plexuses in seen **(B2)**.

To confirm the presence of the four plexuses identified by OCTA, retinal wholemounts from three previously OCTA-imaged eyes were processed for IHC with an antibody (Collagen IV) that labels vessels and imaged by 2-photon/confocal microscopy (Figure 4). A pan-retinal three-dimensional representation of the plexuses and their inter-connections was evaluated, in regions that extended from the ONH to the periphery (*ora serrata*) in each quadrant (Figure 5A and Supplementary Video S1). The SVP contained large arterioles and venules that radiated from the ONH and diverged off, creating tangential branches above and below to form the closely related ICP and RPCP (Figure 5A1). The vessels in the ICP were also seen to branch out and cross through the INL forming the DCP (Figure 5A1,2, white arrows). The DCP is a dense plexus of capillaries and the closest vascular supply to the photoreceptors somatas in the ONL (Figure 4A5). The DCP approaches the SVP towards the periphery (Figure 5A3) to finally merge with it near the ora serrata (Figure 5A4). In the two dense capillary networks (ICP and DCP), intercapillary bridging cells were identified and positively labelled with Collagen IV (Supplementary Video S1). No vascular structures were found beyond the ONL (Figure 4A6).

It is noteworthy that, these four plexuses were not identified in all locations of the retina (Figure 5B1). Peripapillary, the RPCP was only found to extend within 4–5.3 mm from the center of the ONH (Figure 5B2 and Table 4). Although easily identifiable in the central retina, the ICP becomes closer to the SVP in the periphery, precluding its independent visualisation. It was found to merge with the SVP within 9–15.6 mm from the centre of the ONH (Figure 5B and Table 4). In the far periphery, the DCP merged with the SVP (Figure 5B).

**TABLE 4:** Variation in the number of plexuses per quadrant, imaged by 2-photon confocal microscopy.

	Temporal		Nasal		Superior		Inferior	
Plexuses	Mean (*SD*) mm from ONH	Percentage of the total length	Mean (*SD*) mm from ONH	Percentage of the total length	Mean (*SD*) mm from ONH	Percentage of the total length	Mean (*SD*) mm from ONH	Percentage of the total length
RPCP + SVP + ICP + DCP	0–4 (0.5)	33	0–5.3 (0.5)	27	0–4.6 (0.69)	27	0–4 (1)	30
SVP + ICP + DCP	4 (0.5)–9 (1)	41	5.3 (0.5)–15.6 (0.5)	51	4.6 (0.69)–13.6 (0.5)	53	4 (1)–9.5 (1.32)	41
SVP + DCP	9 (1)–12.3 (1.01)	26	15.6 (0.5)–20 (1)	22	13.6 (0.5)–17 (0.6)	20	9.5 (1.32)–13.3 (1.01)	29
Total length	12.3 (1.01)		20 (1)		17 (0.6)		13.3 (1.01)	

*RPCP, radial peripapillary capillary plexus; SVP, superficial vascular plexus; ICP, intermediate capillary plexus; DCP, deep capillary plexus.*

**FIGURE 6:**
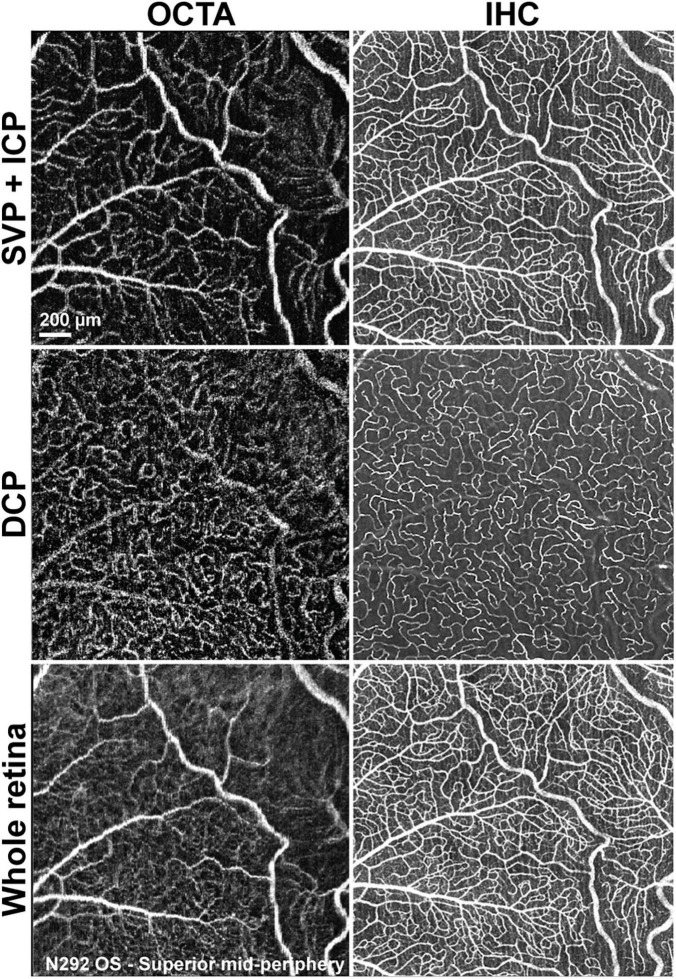
Qualitative comparison of retinal vascular network imaging in dogs by OCTA and IHC. *In vivo* OCTA **(A1,B1,C1)** approaches the level of resolution seen with confocal microscopy/IHC on retinal wholemounts for detection of small capillaries **(A2,B2,C2)**. SVP, superficial vascular plexus; ICP, intermediate capillary plexus; DCP, deep capillary plexus; OS, left eye.

Qualitative comparison of both imaging techniques showed that small capillaries that were well resolved by confocal microscopy/IHC could also be detected by OCTA, albeit with a lower resolution (Figure 6). Images of the SVP + ICP obtained by IHC showed more precisely the smaller capillaries within the ICP, thus resulting in higher VD and JD values in all retinal quadrants, although statistical significance was not always reached, likely due to the small sample size (Supplementary Figure S4). Since the homogeneous ICP was better imaged by IHC than OCTA, LC values were lower in IHC images of the SVP + ICP (Supplementary Figure S4). It is worth noting that small vessels within the DCP were overall well detected with OCTA, and VD values were not found to be significantly different when comparing the two imaging approaches (Supplementary Figure S4). Images from the “Whole retina” slab followed the same trend described for SVP + ICP (Supplementary Figure S4) with a higher VD detected by IHC.

## Discussion

### Optical Coherence Tomography Angiography Reveals Four Vascular Plexuses in the Canine Retina That Share Similarities With Vascular Networks Reported in Human

The retinal distribution of the main arterioles, venules and capillary networks has been previously, albeit imprecisely, described in dogs through the use of vascular corrosion casting combined with light and electron microscopy (Engerman et al., 1966; Flower et al., 1985; Schaepdrijver et al., 1989). However, the number of plexuses, as well as their nomenclature, differs between canine studies (Engerman et al., 1966; Schaepdrijver et al., 1989). With the use of modern imaging techniques, this work has confirmed the presence of four distinct canine retinal plexuses. Since the topographical pattern and location of these plexuses within the retinal layers is analogous to that found in human retinas, we propose that the same nomenclature (RPCP, SVP, ICP, and DCP) outlined by Campbell et al. (2017) be used in all future description of canine retinas (Cuenca et al., 2020).

Similar to the human retinal vasculature, the holangiotic canine retina has a dual blood supply, with oxygenation of the inner retinal layers provided by the four vascular plexuses, and the outer retina relying on the choriocapillaris (Michaelson, 1954). This contrasts with the vascularisation of other vertebrates in which the entire retina relies quasi exclusively on choroidal blood supply (Rochon-Duvigneaud, 1943). Although no studies have reported measurements of intraretinal oxygen tension levels in the dog, it is likely that it shares a similar profile as that of other holangiotic species (Braun et al., 1995). In holangiotic retinas, the outer retinal oxygen levels gradually decrease as a function of distance from the choriocapillaris, and in the inner retina higher levels are found in close proximity to the retinal plexuses, reflecting the dual blood supply. In contrast, in anangiotic or avascular areas of merangiotic retinas, the oxygen values decrease rapidly as the distance from the choriocapillaris increases, reaching very low levels across the majority of the inner retina (Yu and Cringle, 2001). Our detailed analysis of the canine retinal vasculature, particularly the description of the exact location of each plexus within retinal layers, will help improve further understanding of the physiologic and pathologic intraretinal oxygen tension distribution as well as the energy demand and metabolic pathways throughout the various retinal layers in this species.

It is worth noting that while dogs possess a fovea-like region that contains a density of cones comparable to that of primates (Beltran et al., 2014), we did not identify a well-defined foveal avascular zone (FAZ) as described in human eyes (Chui et al., 2012). Nevertheless, in our small cohort of dogs, a poorly defined area with a lower density of vessels was seen in the DCP. Interestingly, this closely resembles the vascular pattern seen in people with a fovea plana. In this condition, the lack of a FAZ and the absence of foveal pit do not affect vision (Marmor et al., 2008; Dolz-Marco et al., 2016; Cicinelli et al., 2017; Chatzistergiou et al., 2021). Hence, the lack of a FAZ in dogs is likely associated with the anatomy of the canine fovea-like area that lacks a foveal pit.

### Optical Coherence Tomography Angiography Can Be Used to Quantify Vascular Parameters in Dogs

Although many studies have reported quantitative data for OCTA images in human retinas, no canine vascular parameters have been described. This study provides normative data for the SVP + ICP, DCP, and WR, successfully establishing a reference range that can be consulted and compared in future studies.

In human eyes, the number of retinal plexuses and their densities diminish toward the periphery (Campbell et al., 2017; Cuenca et al., 2020; Park et al., 2021), and this is similar to what Engerman et al. (1966) previously described in dogs. Our work not only confirms this finding, but now provides quantitative data for four parameters that are frequently used to characterise vascular networks.

Interestingly, we found the VD, JD, and homogeneity (lowest LC) of the DCP to be highest within the *area centralis*. This is very similar to what occurs in the human fovea and this finding indicates that this region of the central canine retina shares closer homology to the primate fovea than previously described (Chan et al., 2012).

Optical coherence tomography angiography signal strength affects the detection of smaller vessels, and Yu et al. (2019) confirmed that a low signal strength can underestimate VD. This highlights the importance of including signal strength or quality parameters when reporting quantitative data (Al-Sheikh et al., 2017). Straightforward ways to optimise quality include, frequently lubricating the ocular surface as well as minimising eye movements through adjustment of the anaesthetic plane to minimise projection and motion artefacts.

### Optical Coherence Tomography Angiography Is a Valid Method for Non-invasive *in vivo* Characterisation of Retinal Vascular Networks in Dogs

We validated the OCTA results by comparison with two other imaging techniques, FA, and IHC (acquired with two-photon/confocal microscopy). The lateral resolution differed between techniques: 5.75 μm/pixel for OCTA (Rocholz et al., 2018), 5.6 μm/pixel for FA and 0.541 μm/pixel for confocal microscopy. The axial resolution of OCTA and confocal microscopy images was 3.87 μm/pixel and 1.5 μm/pixel respectively (Rocholz et al., 2018). All three techniques had sufficient resolution to enable detection of the smallest capillaries, which in our samples had a diameter of approximately 8 μm, similar to that reported in human retinas (Tan et al., 2012). Limitations inherent to each technique explained the differences in image quality but did not affect the quantitative comparison of vascular parameters measured by OCTA and IHC/confocal microscopy.

In our evaluation, OCTA angiograms were of higher resolution when compared to FA images at the same location. Similar to what is reported in humans (Spaide et al., 2015), OCTA in dogs enabled identification of capillary beds (ICP and DCP) that were not identified with FA. However, FA provided a larger field of view and the artefacts that were found in some of the OCTA scans (motion artefacts and decorrelation abnormalities due to projection artefact) were not seen in FA images.

When compared to the images obtained by IHC on retinal wholemounts, our study confirms that OCTA provides good visualisation of the SVP and the DCP. We also found that there was an underrepresentation of small calibre vessels on OCTA, especially those located in highly reflective layers (ICP). These findings have also been reported when evaluating OCTA in *ex vivo* pig eyes (Yu et al., 2021). When compared to images acquired at the same locations by confocal microscopy/IHC, our results suggest that OCTA is a valuable technique for visualising and quantifying retinal vasculature in dogs, especially for analysis of VD in the DCP. Additionally, by IHC we found that the ICP merges with the SVP but not with the DCP as it occurs in human retinas (Chan et al., 2012).

There are some limitations to be considered, such as the small number of eyes that were used for quantitative validation of the OCTA by histology analysis. Additionally, there was minimal misalignment between the images obtained by OCTA and IHC, likely due to shrinkage/expansion due to fixation and distortion of the retinal tissue during the flatmounting process.

## Conclusion

Our study confirmed the feasibility of using OCTA in dogs, providing depth-resolved images from different segmented retinal layers that allow evaluation of individual plexuses. This paves the way for further *in vivo* analyses of canine retinal vasculature in a wide number of retinal pathologies with a vascular phenotype.

## Data Availability Statement

The original contributions presented in the study are included in the article/Supplementary Material, further inquiries can be directed to the corresponding author/s.

## Ethics Statement

The animal study was reviewed and approved by IACUC of the University of Pennsylvania.

## Author Contributions

AR-G conducted all the *in vivo* imaging in the animals, performed the immunohistochemical examination, conceived the project, and wrote the original draft. GR acquired the confocal microscopy images of immunolabeled cryosections. G-SY and YC performed the statistical analysis. NC conceived the project. GA acquired the funding and provided the animal resources. WB conceived the project, acquired the funding, provided the animal resources, supervised the project, reviewed and edited the original graft. All the authors contributed to the article and approved the submitted version.

## Conflict of Interest

The authors declare that the research was conducted in the absence of any commercial or financial relationships that could be construed as a potential conflict of interest.

## Publisher’s Note

All claims expressed in this article are solely those of the authors and do not necessarily represent those of their affiliated organizations, or those of the publisher, the editors and the reviewers. Any product that may be evaluated in this article, or claim that may be made by its manufacturer, is not guaranteed or endorsed by the publisher.

## Footnotes

1
https://ccrod.cancer.gov/confluence/display/ROB2/Downloads
