# Supplementary material for: Characterization of the Canine Retinal Vasculature With Optical Coherence Tomography Angiography: Comparisons With Histology and Fluorescein Angiography
Source: Front Neuroanat. 2021 Dec 13;15:785249. doi: 10.3389/fnana.2021.785249 (PMC8710516; doi:10.3389/fnana.2021.785249)
Supplement: Supplementary file 1 [file Data_Sheet_1.pdf]

SUPPLEMENTARY FIGURES

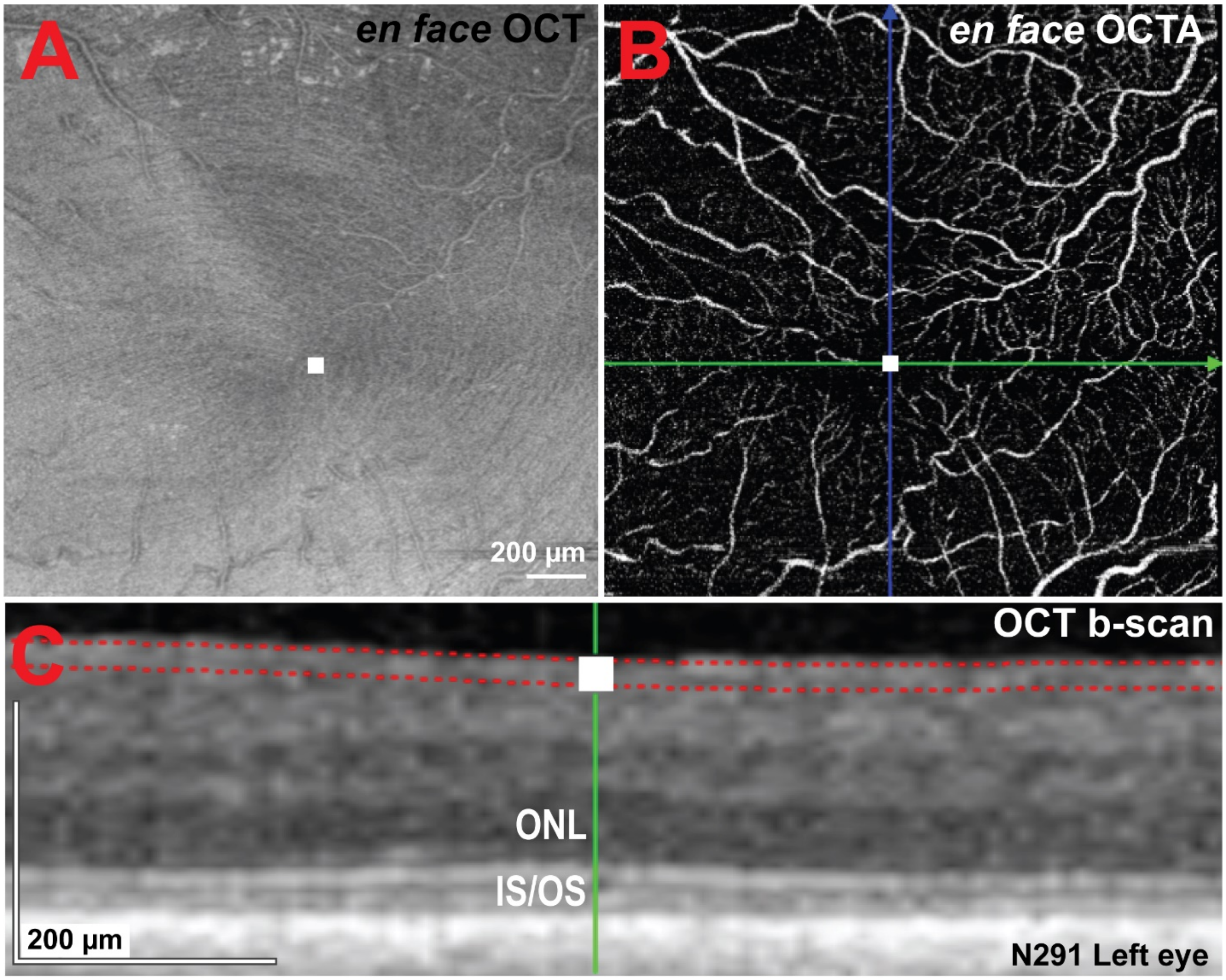

Supplementary Figure S1. Topographical pattern of the nerve fiber layer around the canine fovea-like area. (A) The axons of the ganglion cells bend around the fovea-like area (marked with a white square) when imaged by *en face* OCT. The exact location of the fovea-like area is identified by the converging pattern of the vessels seen by *en face* OCTA **(B)** ONL thinning seen on OCT b scans **(C)**. Dashed lines in (C) represent the slab selected to extract the images in panels (A) and (B).

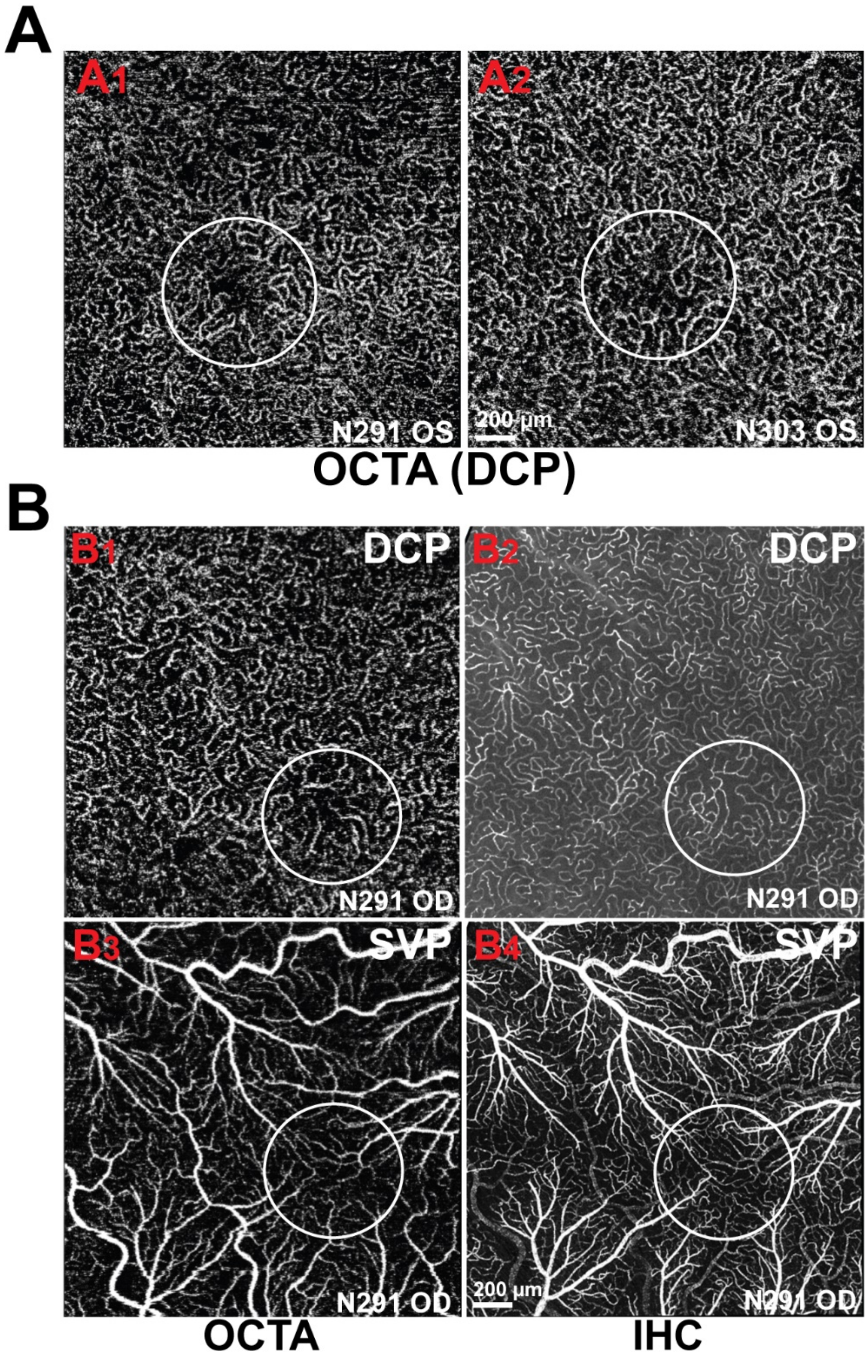

Supplementary Figure S2. Decreased vessel density of the DCP at the canine fovea-like region. (A) OCTA angiograms from two representative animals show a small area with a decreased vessel density of the deep capillary plexus (DCP). **(B)** This feature in the DCP is confirmed by IHC (B_1, B_2). There is no fovea-like avascular zone at the superficial vascular plexus (SVP) level in the same region (B_3, B_4). The white circle shown in all panels in all the images surrounds the canine fovea-like area.

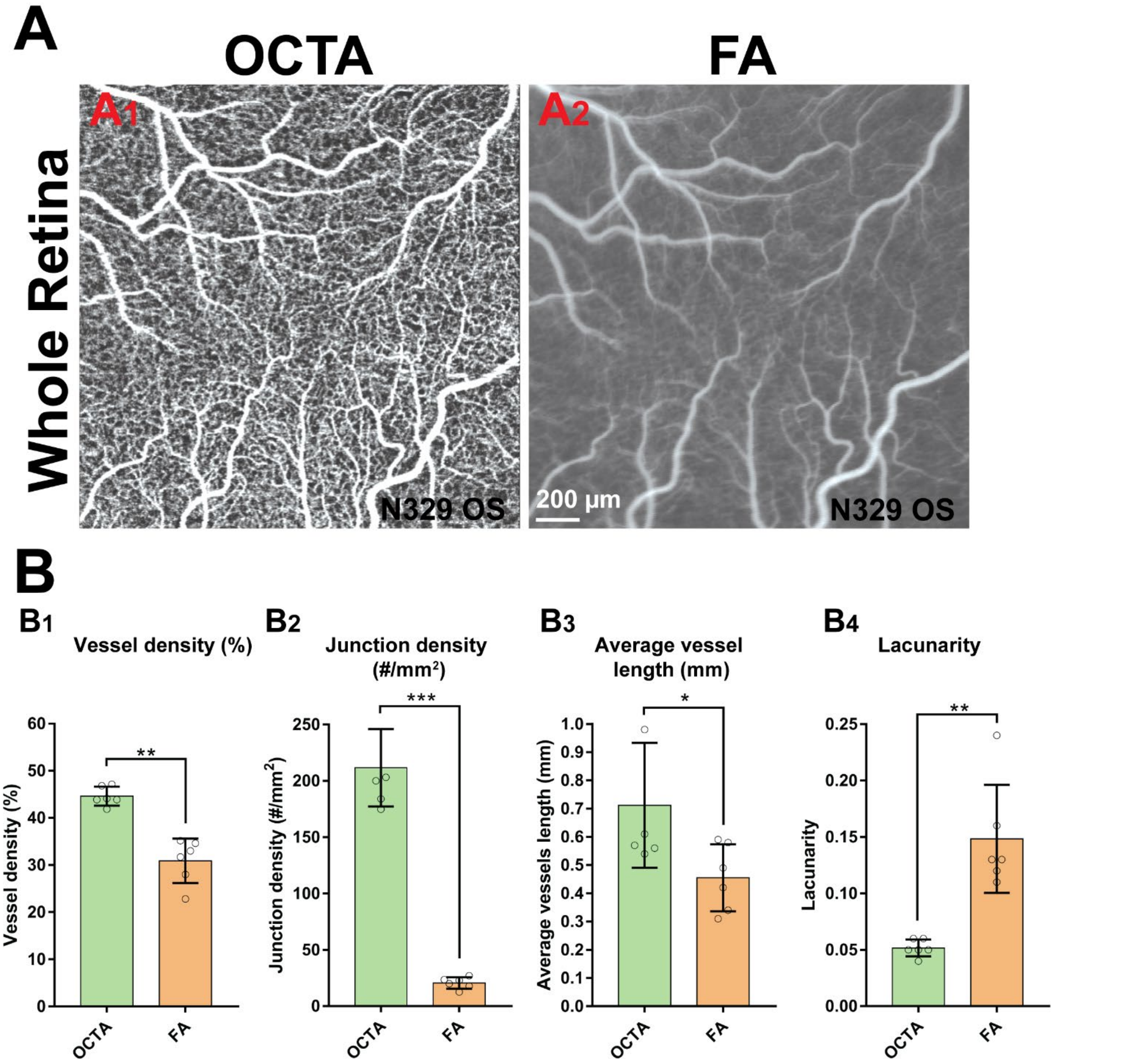

Supplementary Figure S3. Comparison of the canine retinal vasculature imaged with OCTA and FFA. (A) Qualitative comparison of whole retinal thickness angiograms collected by OCTA (A_1) and FFA (A_2) at the same area. **(B)** Quantitative comparison of vascular parameters with both angiography techniques. Results are shown as a mean ± SD ($n = 6$). Paired t-test: *: $p \leq 0.05$, **: $p \leq 0.01$, ***: $p \leq 0.001$.

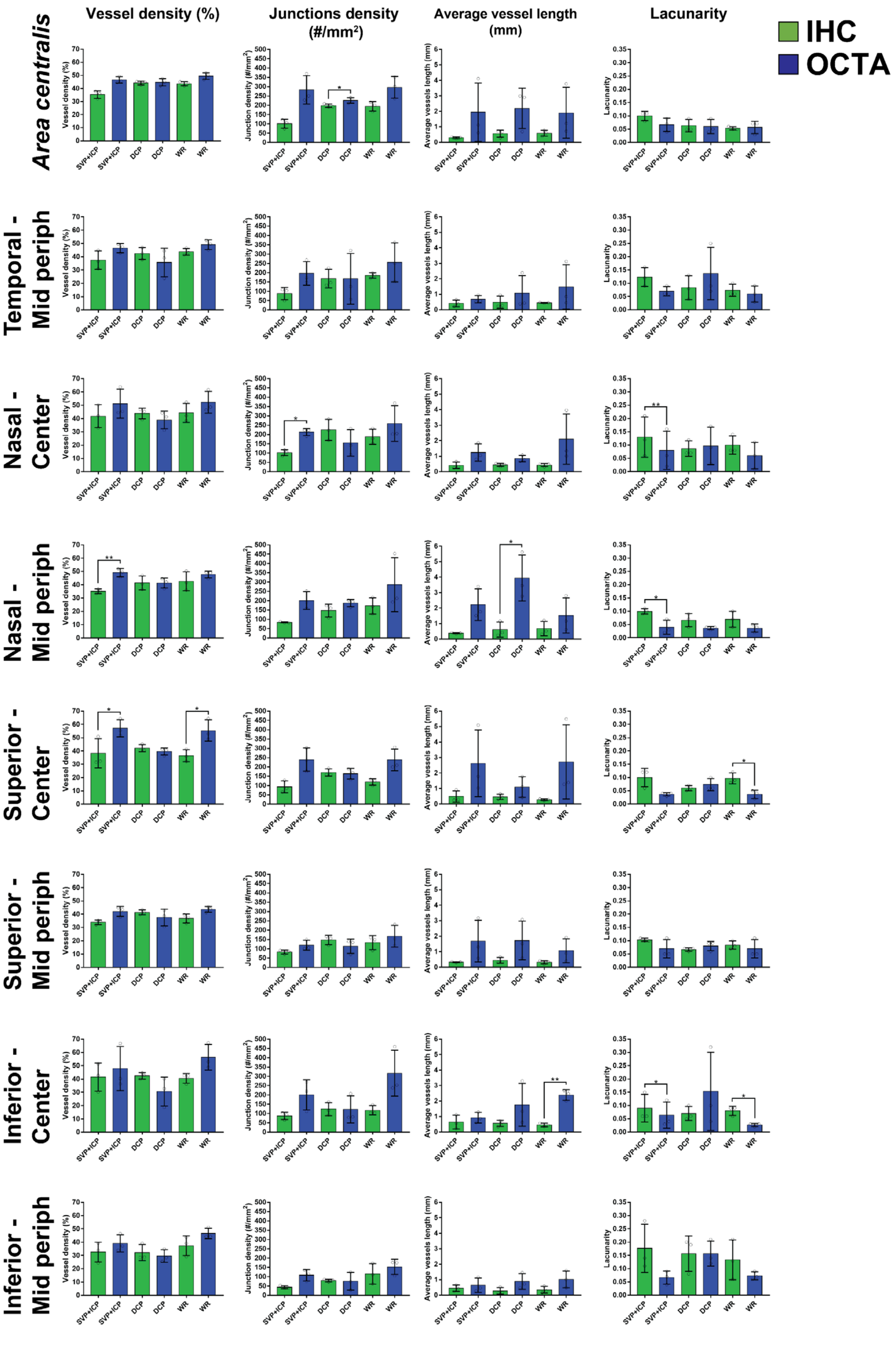

Supplementary Figure S4. Retinal vasculature parameters quantified by OCTA and IHC at the same retinal location and depth. SVP: superficial vascular plexus; ICP: intermediate capillary plexus; DCP: deep capillary plexus; WR: whole retina. Results are shown as a mean ± SD (n = 12). Paired t-test: *: $p \leq 0.05$, **: $p \leq 0.01$, ***: $p \leq 0.001$.

SUPPLEMENTARY TABLE

Supplementary Table S1. Mean quality (signal strength) of the OCTA scans measured in all the retinal areas with the Spectralis HRA/OCT2 unit.

	**Mean OCTA quality in dB (SD)**	
**Area**	**Right eye**	**Left eye**
Area centralis	52.33 (1.51)	52.67 (1.37)
Temporal mid-periphery	48.33 (2.73)	47.83 (4.12)
Nasal centre	48.33 (4.03)	47.5 (2.26)
Nasal mid-periphery	44.5 (2.74)	48.83 (3.71)
Superior centre	47.2 (2.77)	50.83 (2.86)
Superior mid-periphery	43 (4.38)	45.8 (4.44)
Inferior centre	41 (2.83)	42.5 (2.66)
Inferior mid-periphery	35.5 (1.38)	36.83 (3.71)

Note: Signal to noise ratio values provided by the HEYEX Spectralis software range from 20 dB (poor quality) to 53 dB (highest quality).

SUPPLEMENTARY VIDEO

Supplementary Video S1. Topographical patterns of canine vascular retinal plexuses imaged by confocal microscopy (IHC: Collagen IV). Pseudocolored three-dimensional representation of the vascular plexuses imaged by 2 photon/confocal microscopy at 4 locations spanning from the peripapillary region to the to the *ora serrata*. In the peripapillary region, all four plexuses are identified (Radial Peripapillary Capillary Plexus, RPCP; Superficial Vascular Plexus, SVP; Intermediate Capillary Plexus, ICP; Deep Capillary Plexus, DCP). In the central and mid-peripheral retina, the RPCP is no longer seen. At the periphery only the SVP and DCP remain, and both merge at the far periphery close to the *ora serrata*. Intercapillary bridging cells are visualized connecting capillaries, more clearly seen towards the periphery where the vessel density decreases.
